# Clinical implementation of a wide-field electron arc technique with a scatterer for widespread Kaposi’s sarcoma in the distal extremities

**DOI:** 10.1038/s41598-020-66846-5

**Published:** 2020-06-16

**Authors:** Sung-woo Kim, Changhwan Kim, Min-Seok Cho, Seonyeong Noh, Minsik Lee, Chiyoung Jeong, Jungwon Kwak, Minji Koh, Si Yeol Song, Sang-wook Lee, Jeongtae Soh, Seungryong Cho, Byungchul Cho

**Affiliations:** 1 Department of Radiation Oncology, Asan Medical Institute of Convergence Science and Technology, Asan Medical Center, University of Ulsan College of Medicine, Seoul, Republic of Korea; 2 Department of Radiation Oncology, Asan Medical Center, Seoul, Republic of Korea; 3 Department of Radiation Oncology, Asan Medical Center, University of Ulsan College of Medicine, Seoul, Republic of Korea; 4 Nuclear and Quantum Engineering, Korea Advanced Institute of Science and Technology, Daejeon, Republic of Korea

**Keywords:** Sarcoma, Skin cancer

## Abstract

A novel wide-field electron arc technique with a scatterer is implemented for widespread Kaposi’s sarcoma (KS) in the distal extremities. Monte Carlo beam modeling for electron arc beams was established to achieve <2% deviation from the measurements, and used for dose calculation. MC-based electron arc plan was performed using CT images of a foot and leg mimicking phantom and compared with *in-vivo* measurement data. We enrolled one patient with recurrent KS on the lower extremities who had been treated with photon radiation therapy. The 4- and 6-MeV electron arc plans were created, and then compared to two photon plans: two opposite photon beam and volumetric modulated arc with bolus. Compared to the two photon techniques, the electron arc plans resulted in superior dose saving to normal organs beneath the skin region, although it shows inferior coverage and homogeneity for PTV. The electron arc treatment technique with scatterer was successfully implemented for the treatment of widespread KS in the distal extremities with lower radiation exposure to the normal organs beyond the skin lesions, which could be a treatment option for recurrent skin cancer in the extremities.

## Introduction

Kaposi’s sarcoma (KS) is a superficial tumor that typically occurs in the distal extremities of the hands and feet. Surgical excision is not a recommended treatment method for KS because this tumor is spread sparsely on the skin. Systemic chemotherapy is not indicated for the treatment of multiple superficial tumors without distant metastasis, and it is challenging to treat multiple superficial tumors with local cryotherapy^1^. Previous studies have reported that KS responds well to radiation therapy, and so radiation therapy is used to provide palliation with minimal side-effects in all forms of KS^2–5^.

Megavoltage photon beams are generally used to treat widespread skin cancer. These beams have a build-up characteristic near the surface before reaching electronic equilibrium. To treat superficial skin tumors with a megavoltage photon beam, a layer of scattering material that imitates skin called a bolus is used to increase the radiation dose at the skin surface^6^. A previous study has suggested that a simple photon beam technique with various tissue-equivalent boluses such as water or rice could be used in the treatment of skin cancer^7^. This technique has the advantage that it can deliver a uniform dose of radiation to widespread lesions. However, megavoltage photon beam treatment will inevitably deliver as much as the prescribed dose to deep-seated normal tissues. This increases the potential for complications such as fibrosis, edema, and joint contracture as a result of increased radiation exposure to underlying normal tissues such as skin, muscles, vasculature, and bones^3,8^. Electron beam treatments that use beam spoilers could help overcome this issue. Previous studies have confirmed that this technique is useful in treating flat surfaces because the spoiler increases the surface dose and reduces the electron beam’s penetration range^9^. However, because achieving a uniform and homogenous dose distribution using electron beams is challenging on irregular or curved surface lesions, conventional electron beam therapy is a poor option in the treatment of superficial tumors on the hands and feet^10,11^. Therefore, there is a need to develop new radiation therapy techniques to treat superficial tumors while reducing the complications that arise from exposing deep-seated normal tissues that underlie superficial lesions to radiation.

In this study, to overcome the disadvantages of these conventional radiation treatment techniques, we investigated a wide-field electron arc treatment technique with a scatterer that continuously irradiates the electron beam with arc delivery to widespread surface lesions^12^. A conventional electron arc technique is used to treat the chest wall after a mastectomy and has the advantage of avoiding radiation exposure to the normal lung and heart compared to megavoltage photon-beam treatment^13^. Even though dose delivery with an electron arc technique is possible using a commercial linear accelerator (LINAC), dose calculation in this technique is not supported by treatment planning systems (TPSs) due to the computational uncertainty and time consumption. In addition, the irregularity of the treatment surface area and its variable distance from the rotation isocenter compromise the dose coverage of the tumor region and homogeneity of the dose distribution. Therefore, this technique is not widely used.

However, electron arc therapy has a unique benefit in the irradiation of superficial regions while minimizing the radiation dose to deep-seated normal tissues that underlie the superficial target region.

Therefore, in this study, we have developed a novel electron arc treatment technique that incorporates a cylindrical acrylic scatterer that scatters the electrons, thus resulting in a more uniform dose delivery on irregular and curved skin surfaces such as the lower and upper extremities where KS generally occurs.

## Materials and Methods

### Monte Carlo modeling of electron beams

The Tool for Particle Simulation (TOPAS), GEANT4-based MC simulation platform was used to model the 4- and 6-MeV electron beams of a Varian Trilogy linear accelerator^14,15^. The geometry and materials of the components of the Linac head were used to build the Linac model. The beam parameters of the mean energy, energy spread, position spread, and angular spread of incident electron beams to the scattering foil were tuned accurately to achieve agreement within 2% of the measurement results in terms of the Percentage Depth Dose (PDD) and lateral profiles.

### Dosimetric characteristics of the electron arc beams

**Figure 1:**
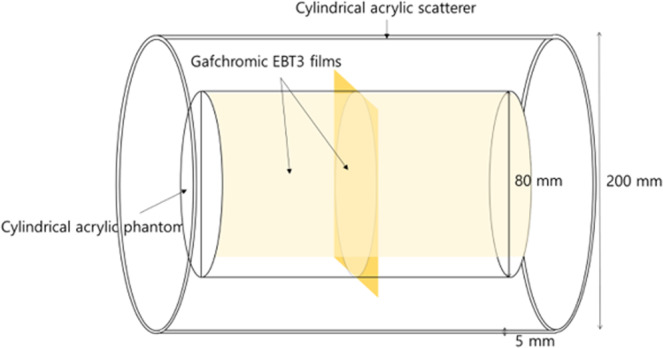
Schematic design for the Monte Carlo simulation and film measurement of the radial and longitudinal dose profiles in the cylindrical acrylic phantom with the scatterer.

Using the established electron beam MC models, electron arc beams were simulated with a modified 25 × 25 cm^2^ electron applicator, the length of which was reduced by 10 cm to avoid collisions with the 20-cm-diameter scatterer during rotation. The radial and longitudinal dose profiles of the electron arc beams were computed in an 8-cm-diameter cylindrical acrylic phantom with 20-cm-diameter and 5-mm-thick acrylic scatterer (Fig. 1). MC simulation was performed with 180 million electrons at 1° intervals to mimic a 360° arc beam delivery.

The radial depth dose and longitudinal dose profile were measured with the modified electron applicator in the same geometry using Gafchromic EBT3 film (Ashland ISP Advanced Materials, USA).

The phantom with the films was positioned at the center of the scatterer and the beam isocenter of the LINAC (Trilogy, Varian, USA). The electron beams (4 and 6 MeV) were irradiated over 360° rotation with the modified electron applicator while preventing collisions with the scatterer. Each electron beam’s longitudinal profile was measured at the surface of the cylindrical phantom. For the treatment of widely spread target longer than 25 cm, the FWHM of the longitudinal profile was measured and tested for field abutting^16^. The radiochromic films were calibrated and analyzed for both the 4- and 6-MeV electron beams^17^.

In addition, the in-air dose profile inside the scatterer was simulated to investigate the variance of the surface dose according to the target’s diameter. Furthermore, narrow-to-wide field sizes were applied to the dose calculation to verify the field size effect. Narrow field sizes of 3 × 25 cm^2^ and 6 × 25 cm^2^ were chosen as they are commonly used to treat the chest wall after mastectomy by conventional electron arc therapy. The chosen wide field size was 20 × 25 cm^2^ as this would fully cover the extremities in the cylindrical acrylic scatterer. MC simulation was performed by delivering 100 million electrons at 1° intervals over 360° for a 4- and 6-MeV electron beam with the scatterer placed at the beam isocenter.

### Phantom study

The feasibility of this technique for widespread lower extremity lesions was investigated with MC simulation and dosimetry measurement using a phantom. To mimic the lower extremities, a foot and leg phantom was made by filling a mannequin made of 2-mm thick transparent plastic with water. The foot and leg phantoms were positioned inside a cylindrical acrylic scatterer that was 20 cm in diameter and 5 mm thick. Planning CT images were acquired using a computed tomography (CT) simulator (Discovery 590 RT, GE Healthcare, USA). The skin region was automatically delineated on the acquired CT images using the Hounsfield Unit (HU) threshold technique over a −800 HU range and the planning target volume (PTV) was delineated up to 5 mm depth from the skin surface. For MC dose calculation, 360 fixed fields were placed at 1° intervals so that the 4-MeV electron beam could deliver a mean dose of 300 cGy to the PTV and mimic the electron arc technique. Two abutting fields were combined with junction match by shifting the distance of the FWHM of the field size. The resulted dose uniformity according to setup variation was checked. Dosimetry measurement using the MOSFET (TN-502RD-HRO, Best Medical Canada, Canada) was performed to measure the surface dose on the phantom at the same geometry as the MC simulation^15^.

### Clinical implementation

This study was reviewed and approved by the Institutional Review Board (IRB) of ASAN Medical center (IRB no. S2020-0671-0001). All procedures were carried out in accordance with IRB guidelines and regulations and informed consent from a patient was obtained. After IRB approval, the developed technique was applied in a patient treated with a fractionated radiotherapy regimen of 30 Gy/10 fx with two opposing field photon beams for KS at the lower extremities 10 years ago but in whom the KS lesions had recurred at the same location^18^. Since radiotherapy with the two opposite photon beam technique delivered the same dose to all structures below the patient’s skin including blood vessels, bone, and muscle tissue, there are special concerns regarding re-irradiating the same lesions. In some cases, normal tissue complications stemming from the radiation of normal tissue has occurred even when using a lower radiation dose than the acknowledged tolerance^19^.

The wide-field electron arc plans at 4 and 6 MeV were created with patient CT images acquired with a cylindrical acrylic scatterer using MC simulation. The treatment field was designed as one full arc over 360° to deliver 3 Gy per fraction at 100% of the PTV, which is sufficient for 5 mm-deep lesions. An acrylic scatterer (20 cm diameter and 5 mm thick) was used to uniformly compensate the electron beam on the skin surface. Two additional treatment plans were created and compared: conventional two opposite photon beams and Volumetric-Modulated Arc Therapy (VMAT) with a 0.5 cm thickness bolus. The two opposite photon and VMAT plans were created using commercial TPS (Eclipse, Varian, USA). In the two opposite photon beam plan, the region in the scatterer on the acquired CT image was removed and a virtual water phantom (30 × 30 × 30 cm^3^) was added to mimic the conventional two opposite photon plan. In the case of VMAT, the region in the scatterer was removed and a 0.5 cm-thick bolus was applied. Plan optimization was performed with an upper dose threshold for normal tissues, bones, and soft tissues. The mean and maximum doses on PTV and normal tissues, conformity index, and homogeneity index for each plan were compared to investigate each plan’s dose characteristics.

## Results

### Monte Carlo modeling of electron beams

**Figure 2:**
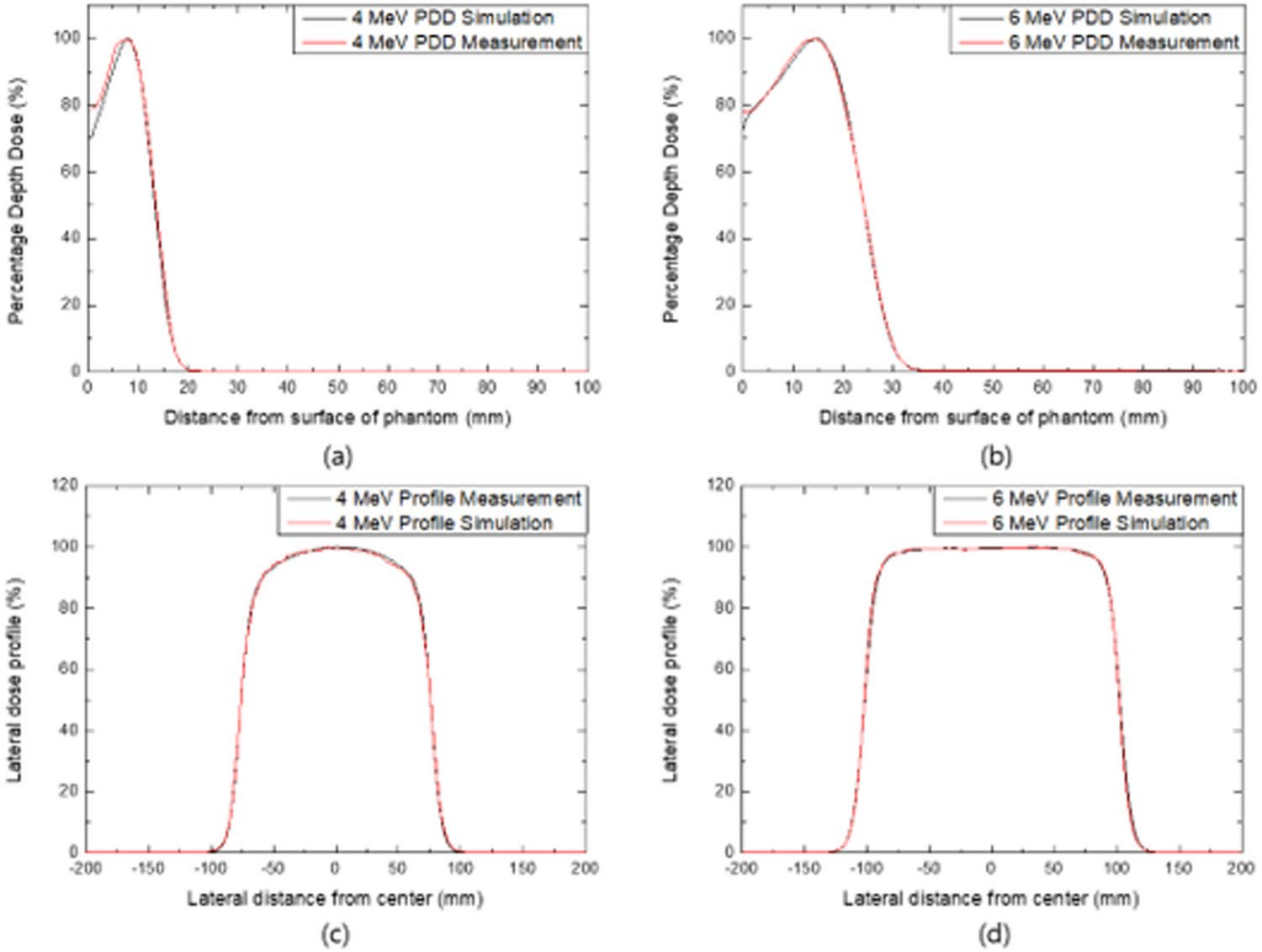
Comparison result of the (**a**) 4-MeV and (**b**) 6-MeV electron PDD, (**c**) 4-MeV, and (**d**) 6-MeV lateral dose profile between the ionization chamber measurement and MC simulation.

Figure 2 shows the comparison results of the MC simulation with the ionization chamber measurement. The PDD of the 4- and 6-MeV electron beams are matched within 0.63% and 0.17% of the average difference, respectively. R_50_ means that the beam quality of the incident electron matches with 0.2 and 0.0 mm difference, respectively. The lateral dose profile at the dose maximum depth for each electron beam is matched with 1.02% and 0.46% average difference and the FWHM of each beam is matched with 0.12 mm and 0.15 mm difference, respectively.

### Dosimetric characteristics of the electron arc beams

**Figure 3:**
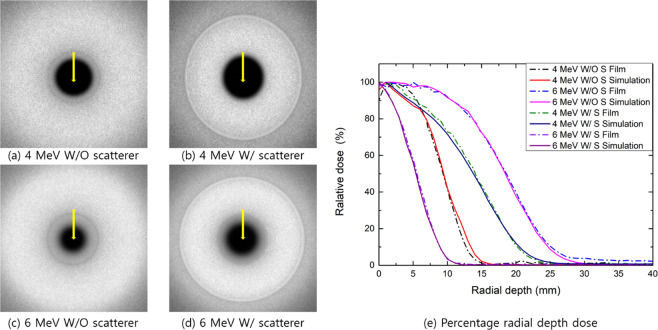
(**a–d**) Simulated images of the electron arc beams with or without the scatterer and (**e**): MC simulation with film measurement comparison results in terms of radial depth dose distributions for the 4- and 6-MeV electron beams with and without the scatterer. The yellow arrows indicate the direction of the radial depth dose.

Figure 3 shows the radial dose profiles achieved with the 4- and 6-MeV electron arc beams with or without the scatterer on the acrylic phantom. The MC simulation results agreed well with the film dosimetry results. The surface doses attained with the 4- and 6-MeV electron beams were >90%, respectively. The radial depth at 80% of the maximum dose was 3.0 and 8.0 mm with the 5-mm acrylic scatterer for the 4- and 6-MeV electron arc beams, respectively. As expected, the range of the electrons was reduced by approximately 5 mm when using the scatterer.

**Figure 4:**
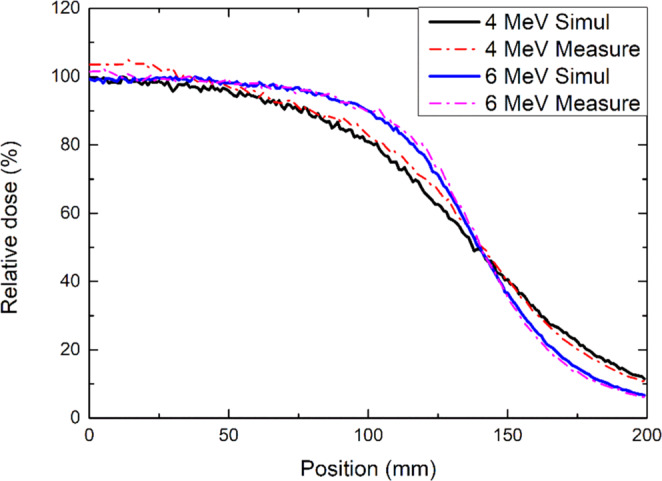
MC simulation and film measurement result of the longitudinal surface dose profile with the acrylic phantom for 4- and 6-MeV electron arc beams.

**Figure 5:**
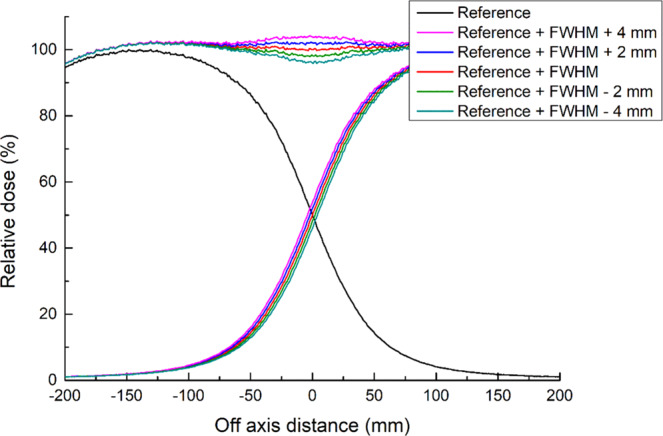
Longitudinal dose profile of two abutting fields with ±4 mm position variation.

Figure 4 shows the longitudinal profiles of the 4- and 6-MeV electron beams. The FWHM of the longitudinal profiles were 27.5 and 28.0 cm, respectively. With respect to the junction treatment, the dose distribution was verified by accumulating the two profiles of the 4-MeV electron beam by moving the profile along the y-axis by 27.5 cm (the FWHM of the longitudinal profile). From the profile accumulation results shown in Fig. 5, the variation in surface dose distribution was ±8% when the position varied by ±4 mm.

**Figure 6:**
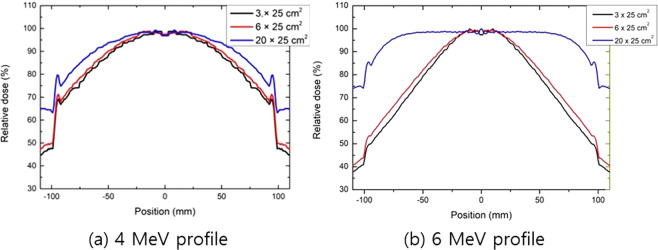
In-air dose profile in the radial direction inside the scatterer for the (**a**) 4- and (**b**) 6-MeV electron beams with various field sizes.

Figure 6 shows the MC simulation results of the in-air dose profile inside the scatterer for various field sizes from narrow to wide. The simulation results show that the in-air dose is maximal at the isocenter and then continuously decreases as it moves close to the inner surface of the scatterer. As the field size changes from narrow to wide, a more homogenous dose was distributed inside the scatterer because more widely scattered electrons would deposit more dose nearer the inner surface of the scatterer. Regarding the electron arc beam with 20 × 25 cm^2^ field size, to cover the target of >90% dose, the skin should be located apart from the inner surface of the scatterer by 2.5 cm or 0.5 cm for the 4- or 6-MeV electron beam, respectively.

### Phantom study

**Figure 7:**
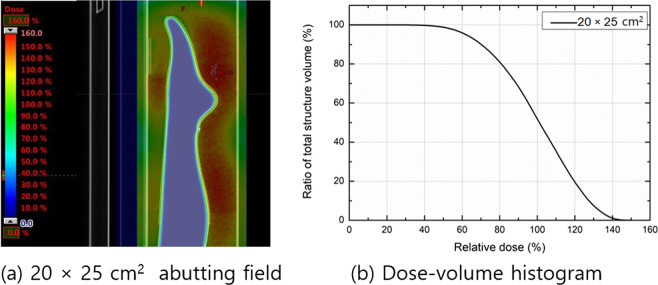
(**a**) The sagittal dose distribution and (**b**) dose–volume histogram of the wide-field (20 × 25 cm^2^) electron arc treatment plan with 4-MeV electron beams. The treatment plans were normalized such that the mean PTV dose was 300 cGy.

Figure 7 shows the results of the feasibility study using the mannequin phantom according to the field size with 4-MeV electron beams. Dose-volume histograms (DVHs) were analyzed for V_90%_, V_100%_, and V_110%_ of the PTV; these were 68.3, 52.0, and 35.5%, respectively.

**Figure 8:**
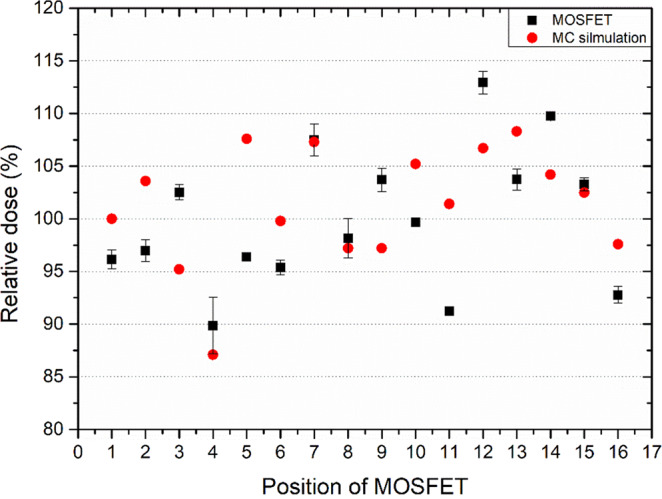
MC-computed and measured surface dose for a foot and leg phantom filled with water to verify the surface dose uniformity.

As shown in Fig. 8, the MC–computed and MOSFET–measured surface dose measured were delivered at around ±10% of the prescribed dose, which shows fairly good uniformity.

### Clinical implementation

**Figure 9:**
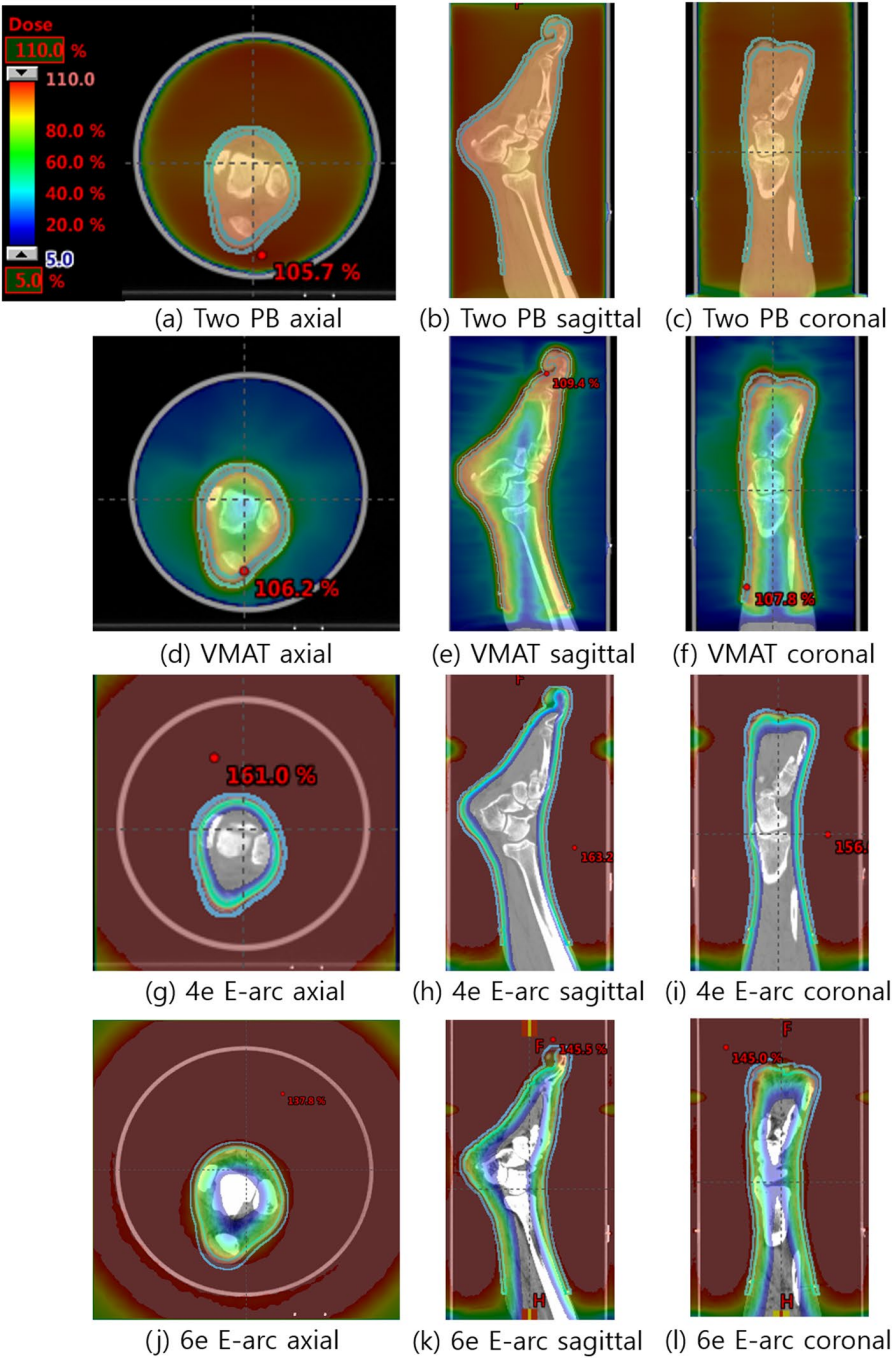
Axial, sagittal, and coronal views of the dose distribution in the three different plans. (**a–c**) Two opposite photon plan, (**d–f**) VMAT plan with bolus, (**g–i**) 4-MeV field abutting plan with electron arc technique and (**j–l**) 6-MeV field abutting plan with electron arc technique.

**Figure 10:**
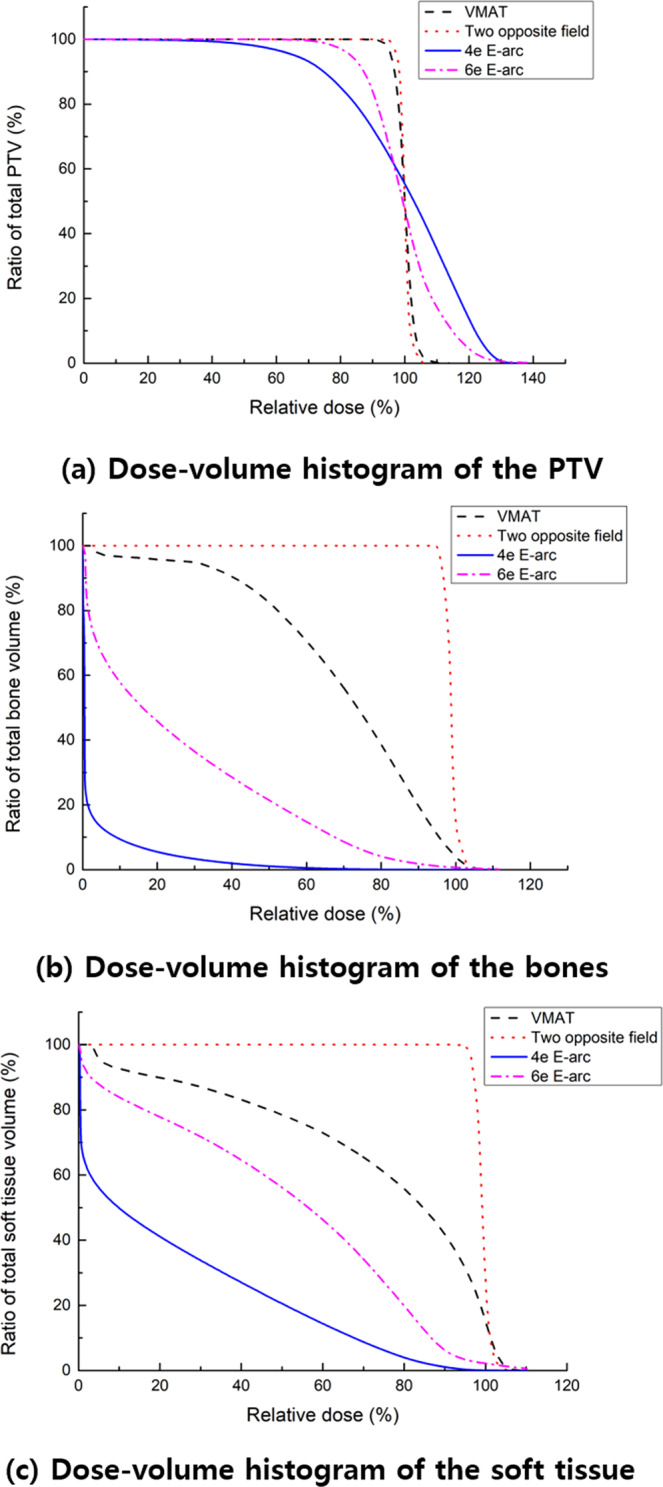
The Dose–volume histograms of widespread Kaposi’s sarcoma on lower extremities. (**a**) PTV, (**b**) soft tissue, and (**c**) bones from two opposite photon, VMAT, and electron arc (E-arc). When using the electron arc plan, the maximum dose to PTV was higher than the other photon plans. However, the dose delivered to underlying normal organs, soft tissues, and bones were dramatically decreased when using the electron arc plan.

Figure 9 shows the dose distributions of the four different treatment plans, Fig. 10 illustrates the DVHs of the PTV and OARs, and Table 1 summarizes the dosimetric results from each plan. The D_2%_ and D_98%_ were analyzed to investigate the maximum and minimum dose on the target volume. The D_2%_ was highest for the 4-MeV electron arc plan with a value of 38.57 Gy. The D_98%_ was lowest at the 4-MeV electron arc plan with a value of 15.78 Gy. Therefore, the homogeneity index (HI) was highest at the 4 MeV electron arc plan with value 2.44 and the conformity index (CI) was lowest at the electron arc plan with value 0.65. However, the mean dose to OARs, soft tissue, and bones, were lowest for the 4-MeV electron arc plan with values 7.05 and 1.03 Gy, respectively.

**Table 1:** Dosimetric results of the four treatment plans.

Treatment Plan	Two opposite photons	VMAT	4-MeV electron arc	6-MeV electron arc
Target mean dose (Gy)	30.00	30.00	30.00	30.00
Target maximum dose (Gy)	31.84	34.20	41.54	47.64
D_2%,PTV_ (Gy)	30.85	31.72	38.57	37.16
D_5%,PTV_ (Gy)	30.85	31.26	37.62	35.83
Target minimum dose (Gy)	26.41	24.23	0.34	15.15
D_98%,PTV_ (Gy)	28.90	28.32	15.78	23.35
D_95%,PTV_ (Gy)	29.24	28.72	19.50	24.97
HI (D_2%,PTV_/D_98%,PTV_)	1.12	1.08	2.44	1.59
HI (D_5%,PTV_/D_95%,PTV_)	1.09	1.06	1.93	1.44
CI (V_95%_/Target volume)	0.99	0.97	0.65	0.67
Maximum dose to soft tissue (Gy)	31.84	34.06	33.06	39.48
Mean dose to soft tissue (Gy)	29.76	21.94	7.05	15.3
Maximum dose to bones (Gy)	31.52	33.52	30.62	36.21
Mean dose to soft bones (Gy)	29.63	20.96	1.03	7.8

Compared to the two photon techniques, the electron arc plans resulted in superior dose saving to normal organs beneath the skin region, although it shows inferior coverage and homogeneity for PTV. With a special concern of re-irradiation for the patient, the 4 MeV electron arc beam was preferred to minimize radiation exposure to normal structures underneath the treatment lesion. The complete response rate of cutaneous KS for the prescribed dose difference was previously reported as 40 Gy (83%) and 20 Gy (79%)^18^.

The KS lesions were spread over the patient’s toes and calves over >40 cm. To treat this large recurrent lesion, a treatment plan using the wide-field electron arc technique with a scatterer was established by overlapping two fields with a junction at the shin. To fix the treatment lesion inside the scatterer without interactions with the electron beam, low-density Styrofoam was used as supporting material. The scatterer was used as an immobilization device to ensure setup reproducibility.

The treatment proceeded as follows. First, the cylindrical scatterer was attached to the front of the treatment couch to minimize electron scattering from the treatment couch. The region being treated was placed into the fixed scatterer. The junction position at the shin was aligned to the beam isocenter of the LINAC using an in-room laser system. An on-board imaging (OBI) system was used for kV image guidance to ensure inter- and intra-fractional setup reproducibility^20^.

**Figure 11:**
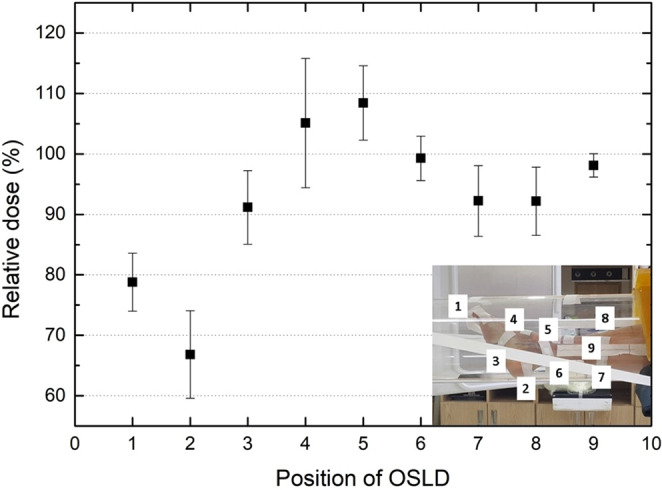
*In vivo* dosimetry using OSLD to re-irradiate widespread recurrent KS using an electron arc technique with a scatterer.

To deliver the prescribed dose uniformly to the entire treatment lesion, the treatment was delivered to each abutting field by moving the couch a full width at half maximum (FWHM) of the electron beam’s longitudinal profile. The total treatment time, including setup, kV image acquisition, and beam delivery, was within seven minutes, including two minutes of irradiation time. *In-vivo* dosimetry using an OSLD system was performed to verify the precise delivery of electrons according to the established treatment plan^21^. It showed that the surface dose was within 10% of the prescribed dose except at positions 1 and 2 (the toe and heel), which were located near the scatterer (Fig. 11).

## Discussion

This study investigated an electron arc treatment technique with scatterer to treat widespread skin cancer and applied it in a patient who was re-irradiated at the lower extremity for recurrent KS. Compared with other radiation treatment techniques, this technique is advantageous when treating widespread skin cancer on the arms and feet with reasonable dose homogeneity while reducing the dose absorbed by the underlying normal tissues beyond a skin such as blood vessels and bone. A plan comparison study to treat multifocal KS on the foot using various radiation treatment techniques such as multi-field electron therapy, brachytherapy, intensity-modulated radiation therapy (IMRT), and volumetric arc therapy (VMAT) was reported by Park *et al*.^22^. Electron beam therapy has the disadvantage of requiring many treatment fields for multifocal lesions, which could cause uncertainty in patient positioning, long treatment time, and difficulties in field matching. High-dose-rate (HDR) brachytherapy with surface applicators has the disadvantage of small treatment window and the need for large, flexible boluses. VMAT has advantages for the treatment of multifocal lesions in terms of target conformity, homogeneity, and the reduced delivery of radiation to normal organs compared to HDR and conventional electron techniques. However, VMAT results in the delivery of approximately 30% of the target mean dose to deep-seated structures, including bone.

In this study, we developed and evaluated a novel wide-field electron arc technique to overcome the disadvantages of previous techniques. Our method has several advantages for the treatment of widespread skin cancer on cylindrical regions such as the arms and legs.

First, the developed technique is more effective for dose saving on normal organs underlying skin lesions. As expected, the previously reported plan comparison study and plan comparison result in this study shows that radiation exposure to underlying normal tissues is unavoidable when treating widespread skin lesions with a photon beam. Although the developed electron arc technique with a scatterer has less conformity and homogeneity than the conventional photon plans, it has the unique advantage that it saves the underlying normal organs with reasonable PTV coverage and homogeneity. This advantage makes this technique a superior candidate treatment method for widespread recurrent skin cancer, which is a special concern in radiation exposure to save the normal underlying organs on the arms and legs.

Second, the delivery of radiation to widespread skin lesions using this technique is reasonably uniform. With a scatterer, generated electrons from the LINAC are scattered, and these scattered electrons are deposited uniformly across wide skin lesions on cylindrical feet or arms. When using a scatterer, if the field size is not sufficiently large, the interaction area of the electron beam with the scatterer will be relatively small, and a smaller amount of electrons will be scattered. Therefore, if the treatment lesion is irregular and larger than the used field size, the dose will be distributed unevenly across the treatment lesion^23^. Therefore, when using an electron arc treatment with a scatterer for lesions on the arms and legs, the field size must be sufficiently large to cover the scatterer to deliver a uniform dose due to the wide field electron beam making more scattered electrons with acrylic scatterer.

Third, the developed techniques have the advantage of treatment convenience in the treatment set-up compared to conventional photon plans. When using a two opposite photon and VMAT plan, build-up materials such as water, rice, and bolus, are essential for delivering sufficient radiation to the patient’s skin. However, these methods are very labor-intensive and setting up a bolus without an air-gap and slipping it down on the wide region of the patient’s curved skin are difficult. These issues make dose uncertainties when treating lesions and this may repeat in every treatment fraction. When using the isocentric electron arc technique with a scatterer on cylindrical treatment lesions, the delivered doses are even for the same distance from the center of the scatterer. Therefore, it could provide more convenience in the treatment set-up and accuracy of inter-fractional treatment than conventional photon plans. The total treatment time—including patient setup with kV image and beam delivery—was approximately seven minutes, which is a significant improvement compared to treatment with multiple patched electron beams, which requires insertion/removal procedure for the beam shaping block and is equivalent to treatment with IMRT/VMAT with KV image guidance. In addition, our method may increase patient safety during radiation treatment because the insertion and removal procedure when shaping applicators into the LINAC gantry are removed when the patient lies on the treatment couch.

This electron arc technique could be useful for treating skin cancer patients who have received prior radiotherapy in whom it is particularly important to minimize the radiation exposure of normal tissues of the irradiated arms and legs.

One limitation of this study is that the radiation dose decreases near the scatterer. The MC simulation and dosimetry measurement showed that deposited electrons continuously decreased to 75% of the maximum absorbed dose at the inner surface of the scatterer, even when using a wide field electron beam. This phenomenon is due to the loss of energy of electrons passing through the scatterer and being deposited at its inner surface. This makes the uniform delivery of the prescribed dose to the treatment lesion difficult. However, this could be mitigated by using low-density materials as an immobilizer to separate the skin target of the feet/legs from the inner space of the scatterer by 0.5–2.5 cm.

## Conclusion

An electron arc treatment technique with external scatterer for the treatment of KS on the skin of the foot was evaluated and applied clinically. This technique could be useful for treating multifocal skin diseases including cutaneous KS on the arms and feet.

## Data Availability

The datasets generated during and/or analysed during the current study are available from the corresponding author upon reasonable request.
